# Biostimulation of Tomato Plants (*Solanum lycopersicum* L.) Using Fragmented Extracellular DNA from *Clavibacter michiganensis*

**DOI:** 10.3390/plants15111599

**Published:** 2026-05-22

**Authors:** Ireri Alejandra Carbajal-Valenzuela, Luz María Serrano-Jamaica, Lucía Vazquez, Gabriela Medina-Ramos, Ramón Gerardo Guevara-González

**Affiliations:** 1 Center of Applied Research in Biosystems (CARB-CIAB), Autonomous University of Querétaro, Amazcala, El Marques, Queretaro 76265, Mexico; ireri.carbajal@uaq.mx; 2 Molecular Plant Pathology Laboratory, Polytechnic University of Guanajuato, Cortazar 38496, Mexico; rgggon@hotmail.com; 3 Biology Department, University of Illinois Springfield, Springfield, IL 62703, USA; lvazq1@uis.edu

**Keywords:** bioestimulation, elicitors, extracellular DNA, PAMPs

## Abstract

Extracellular DNA (eDNA) has gained attention as a danger signal between organisms because of the ecological implications of this mechanism and its great potential as a biological modulator in agriculture. Self-DNA and non-self DNA have been evaluated earlier, both as plant immune system elicitors. Here we show the effect of eDNA extracted from the bacterial phytopathogen *Clavibacter michiganensis* applied to tomato plants in different concentrations (50, 100 and 150 µg mL^−1^). Monitoring morphology of the plants, spectrophotometric determinations and RT-qPCR assays showed a dose-dependent effect on plant growth and root development, activation of antioxidant enzymes such as catalase and superoxide dismutase, biosynthesis of secondary metabolites, including phenolic compounds and flavonoids, and differential expression of genes related to plant stress response, such as chalcone synthase and phenylalanine ammonia-lyase. Lower concentration treatments showed an increment in the variables as beneficial responses for agricultural practices, and the higher concentration (150 µg mL^−1^) showed reduced or no effects on the evaluated variables. This work represents a step forward in the development of effective and more sustainable agricultural technology in crop production.

## 1. Introduction

Plants’ survival depends mostly on their capacity to sense and understand their close surroundings and display the right responses on time and intensity to overcome potential dangers [1]. Their immune systems rely on the structures that coevolution has shaped according to the most important signals to detect, for example, the presence of microbial pathogens, and once the signals have been sensed, a specific signal transduction starts, including several cascades of molecular events generally ending in expression changes in genes [2]. The defense program that is activated depends on the signals that the system detects, one of the commonly reported is the PAMP-triggered immunity (PTI), which is sensitive to pathogen (or microbial)-associated molecular patterns (PAMPs) and consists of the initial general layer of defense whose main objective is to restrict the pathogen proliferation inside the plant tissues [3]. PAMPs have been reported as highly conserved molecules in common pathogen structures, sensed by organisms as danger signals [4]. Some molecules identified as PAMPs are flagellin, a ubiquitous component of bacterial flagella that causes the activation of early immune signals in plants. Such signal activation includes receptor-like cytoplasmic kinases (RLCKs), ion channels and defense-specific mitogen-activated protein kinases (MAPKs), accumulation of reactive oxygen species (ROS), and an increase in cytosolic Ca^2+^ influx, among other things that eventually end in the synthesis of secondary metabolites and defense hormones [5]. Another PAMP example is the elongation factor Tu (EF-Tu), one of the most abundant bacterial proteins that activate the so-called PAMP-triggered immunity (PTI) in plants. EF-Tu causes the transcriptional reprogramming of defense genes such as WRKY transduction factors and pathogenesis-related proteins (PR), different from the activation of the effector-triggered immunity (ETI) response [6]. A recently identified PAMP is the extracellular DNA (eDNA). eDNA has been addressed as a damage-associated molecular pattern (DAMP) when it is sensed as self-DNA in multiple organisms [7], but recent studies have also shown that plants respond to eDNA extracted from potential pathogens (i.e., as PAMP). Serrano-Jamaica et al. [8] applied a mixture of fragmented DNA extracted from common phytopathogenic microorganisms (*Phytophthora capsici*, *Fusarium oxysporum* and *Rhizoctonia solani*) to *Capsicum annumm* plants and reported the activation of several defense responses, such as an increment of phenolic compounds and total flavonoids, and upregulation of phenylalanine ammonium lyase and chalcone synthase. Moreover, as proof of immune activation, the treated plants showed more resistance to the induced infection of these three pathogens at the same time.

Finally, the role of eDNA as part of a strategy to manage tomato (*Solanum lycopersicum*) pathogenic fungus *Fusarium oxysporum* has been addressed as a DAMP while applying eDNA from the plant and as a PAMP when applying eDNA from the pathogen [9], opening the possibility of an eDNA-based agricultural application. *Clavibacter michiganensis* (*Cm*) is a Gram-positive phytopathogenic bacterium that causes bacterial canker in tomato with devastating nature, along with losses of millions of dollars each year [10]. *Cm* infects the xylem vessels and causes symptoms of wilting, marginal leaf chlorosis and cracks on the stem [11]. The infection can access seeds through the xylem but also externally through tomato fruit lesions, which makes the control and management of the disease difficult [12]. Currently, there are no commercial fully resistant cultivars, so successful and sustainable management approaches need to be developed, such as the controlled application of plant elicitors like PAMPs that trigger immunity in crops.

Aside from the elicitation function that eDNA performs in the plant’s immune system, the effect on variables of agricultural interest, such as growth and development, known as biostimulation, has not been fully evaluated. This trait should be addressed in PTI activation events to avoid a negative impact on agronomical variables of the culture. Biostimulants have an effect on the primary metabolism of plants and cause an increment in plant growth and development through the plant phenological stages [13]. This may finally be reflected in yield increments as well as in the whole plant performance. Thus, based on the aforementioned scenario, this work aimed to evaluate the biostimulation effect of tomato (*Solanum lycopersicum*) using extracellular DNA fragments of the pathogen *Cm* as an elicitor.

## 2. Results

### 2.1. Effect of Applying eDNA Fragments from Cm on Morphological Variables in Tomato Plants

**Figure 1:**
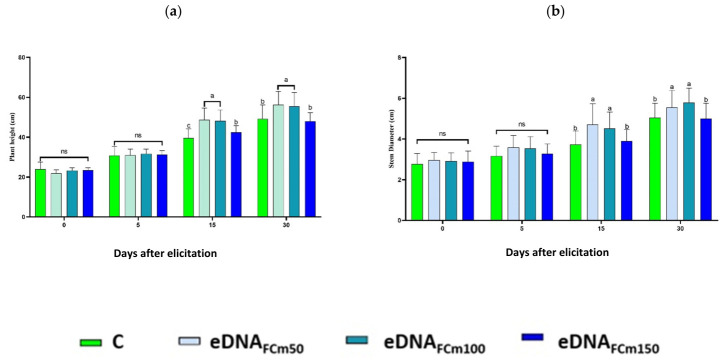
Morphological variables in tomato plants. (**a**) Plant height and (**b**) Stem diameter evaluated at different times (0, 5, 15 and 30 dae). Two-way mean ± standard deviation, Tukey’s test, *p* < 0.005. Significant differences between treatments are indicated by different letters. C: Control plants. FCm: DNA fragments from the pathogen *Cm*; 50, 100, and 150, concentration of eDNA fragments in µg mL^−1^. Ns: not significant difference.

Plant height and stem diameter were determined at 0, 5, 15, and 30 days after elicitation (dae) with eDNA of *Cm*. The height of plants treated with eDNA fragments of the *Cm* is shown in Figure 1a. A statistically significant increase in plant height was observed at 15 and 30 dae in treatments with eDNA fragments of the *Cm* at concentrations of 100 (_FCm100_) and 150 (_FCm150_) µg mL^−1^, compared to the control plants (C).

The measurement of a plant’s stem diameter is a fundamental variable for assessing plant health, monitoring its development, and even planning its management and harvest. It indicates the strength and vigor of the plant’s growth, affects its capacity to support and transport fluids (water and nutrients), and can be a predictor of crop quality and yield [14]. At 15 and 30 dae, a statistically significant increase in diameter was observed for eDNA_FCm50_ and eDNA_FCm100_ treatments compared to C (Figure 1b).

**Figure 2:**
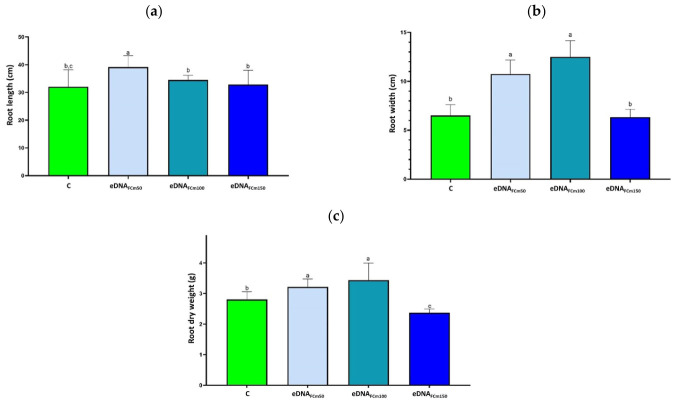
Morphological variables in the root of evaluated tomato plants. Length (**a**), width (**b**), and dry weight (**c**) of roots at 30 dae. Two-way mean ± standard deviation, Tukey’s test, *p* < 0.005. Significant differences between treatments are indicated by different letters. C, Control plants. FCm, DNA fragments from *Cm*; 50, 100, and 150, concentration of the eDNA fragments given in µg mL^−1^.

Another important organ for the survival and healthy growth of plants is the root; the root size (length, width, and dry weight) is related to the search for resources in the soil, thereby determining the plant’s ability to absorb water and nutrients and providing physical anchorage [15]. In herbaceous plants, Wijesinghe et al. [16] found that root size was related to the accuracy of nutrient search among species; root system size is also related to the yield and drought tolerance of many plants. The length, width, and weight of the roots were evaluated at 30 dae as indicators of healthy growth in tomato plants (Figure 2). In the case of root length (Figure 2a), a statistically significant increase was observed among the eDNA_FCm50_ treatment plants compared to C. The highest values for root width and dry root weight were obtained for eDNA_FCm50_ and eDNA_FCm100_ treatments, showing a statistically significant difference compared to C and eDNA_FCm150_ treatments (Figure 2). In dry root weight, a decrease was observed as an effect of the higher concentration of eDNA (150 µg mL^−1^).

**Figure 3:**
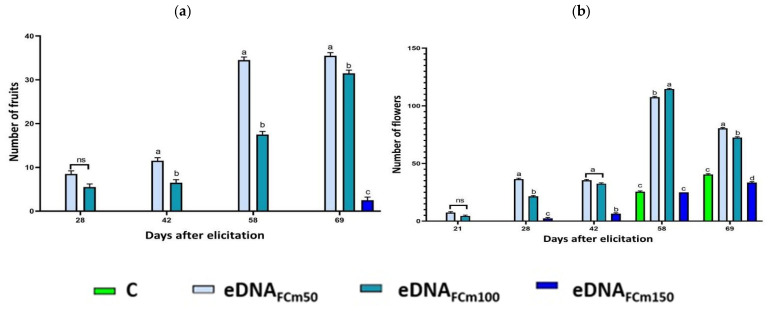
Flowering and fructification of tomato plants. Panel (**a**), number of fruits per plant; panel (**b**), number of flowers per plant. Significant differences between treatments are indicated by different letters. C, Control plants. FCm, DNA fragments from the *Cm*; 50, 100, and 150, concentration of the eDNA fragments in µg mL^−1^.

Phenological variables were also measured as indicators of the biostimulant effect in plants; Figure 3 shows the number of flowers (a) and fruits (b) present in the plants at different times of observation. On the one hand, in plants treated with eDNA_FCm50_ and eDNA_FCm100_ treatments, flowers were observed since day 21 after elicitation, reaching a sum of more than 100 flowers at the end of the assay. In contrast, plants treated with eDNA_FCm150_ produced the first flowers at 28 dae and showed a slow rate of flowering compared with other treatments. Finally, control plants showed their first flowers on day 59 after elicitation, but reached a total number of flowers higher than treatment eDNA_FCm150_ on day 69 after elicitation. On the other hand, the number of fruits per plant was consistently higher in treatments with eDNA_FCm50_ and eDNA_FCm100,_ with eDNA_FCm50_ being significantly the best from 42 to 69 dae (Figure 3b). Interestingly, treatment eDNA_FCm150_ displayed a few numbers of fruits at 69 dae; additionally, in C, no fruits were detected at least until 69 dae evaluated in the study. This latter phenotype might be caused by some environmental conditions during the study, an aspect that the fragmented *Cm* eDNA (especially at 50 and 100 µg mL^−1^ treatments) helped to overcome and biostimulate this variable for fruit production in this tomato cultivar under the conditions used in the study.

**Figure 4:**
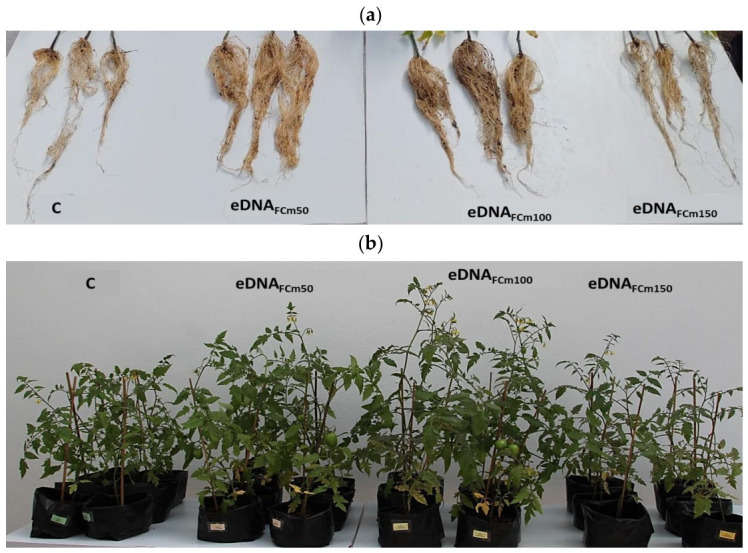
Root and aerial parts of tomato plants evaluated at 30 dae. C, Control plants. FCm, DNA fragments from the *Cm*; 50, 100, and 150, concentration of the eDNA fragments in µg mL^−1^.

Figure 4 shows the phenotypic characteristics of tomato plants subjected to treatments with eDNA fragments from *Cm*, as well as C at 30 dae. Roots (Figure 4a) showed higher biomass density production on 50 and 100 µg mL^−1^ concentrations of the treatment. Aerial parts (Figure 4b) showed higher stems and the presence of fruits and flowers in these treatments. This was not observed at plants treated with eDNA_FCm150_ treatment, which showed a phenotype more like C.

### 2.2. Total Phenol and Flavonoids Levels in Tomato Plants Treated with the eDNA Fragments from Cm

Plants produce substances that act as a first line of defense when subjected to biotic or abiotic stress, initiating the synthesis of phenolic compounds and flavonoids in a short time once the stress is detected [17]. The production of phenolic compounds in plants works against oxidative damage, while the production of flavonoids has been directly linked to protection against pathogens and drought [18].

**Figure 5:**
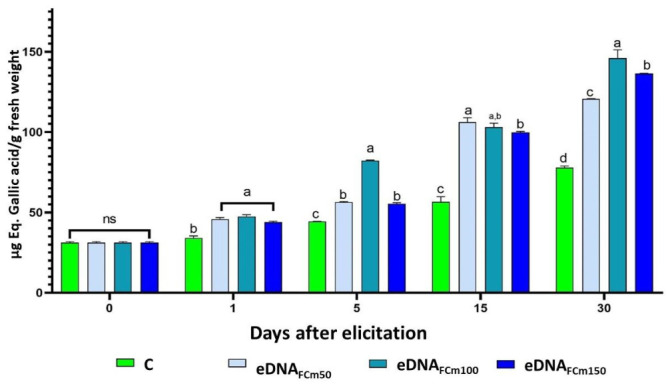
Total phenols from plants treated with eDNA fragments from *Cm*. Samples at 0, 1, 5, 15, and 30 dae. C, Control plants. FCm, DNA fragments from *Cm*. 50, 100, and 150 concentrations of the eDNA fragments are given in µg mL^−1^. Statistical analysis was performed using two-way ANOVA, Tukey’s test, *p* < 0.05. Significant differences between treatments are indicated by different letters.

The results obtained in the different treatments at 0, 1, 5, 15, and 30 dae showed an increase in the concentration of these phytochemicals (Figure 5). This increase was gradual over time in all plants, but it was significantly the highest level in plants treated with eDNA_FCm100_.

**Figure 6:**
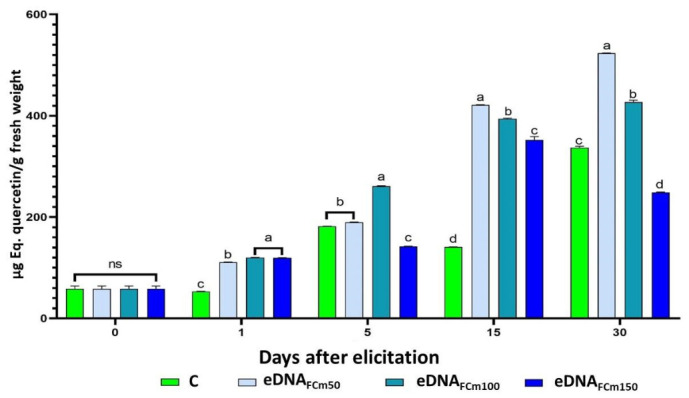
Total flavonoids from plants treated with eDNA fragments from *Cm*. Samples at 0.1, 5, 15, and 30 dae. C, Control plants. FCm, DNA fragments from *Cm.* 50, 100, and 150 concentrations of the eDNA fragments are given in µg mL^−1^. Statistical analysis: two-way ANOVA, Tukey’s test, *p* < 0.05. Averages with identical letters indicate no statistically significant differences (ns).

In the case of total flavonoids production (Figure 6), at 1 dae, the highest concentration was found in the eDNA_FCm100_ and eDNA_FCm150_ treatments. At 5 dae, the concentration increased for all treatments, with the highest level observed for the eDNA _FCm100_ treatment. At 15 dae, the concentration of total flavonoids increased for all treatments with eDNA from the pathogen *Cm*, but not for C. At 30 dae, the highest concentrations were found in the eDNA_FCm50_ treatment. Noteworthy mentioning that the higher flavonoid content compared to total phenolics is due to differences in the assay sensitivity and the use of different reference standards (quercetin vs. gallic acid) for these measurements, which are not directly comparable to each other. However, the trends observed for the contents of both types of secondary metabolites used in plant defense after treatments with FC eDNA treatments (especially at 50 and 100 µg mL^−1^ were significantly increased in comparison to C (Figure 5 and Figure 6).

### 2.3. Analysis of Gene Expression Associated with Stress Response in Tomato Plants Treated with the eDNA Fragments of Cm

**Figure 7:**
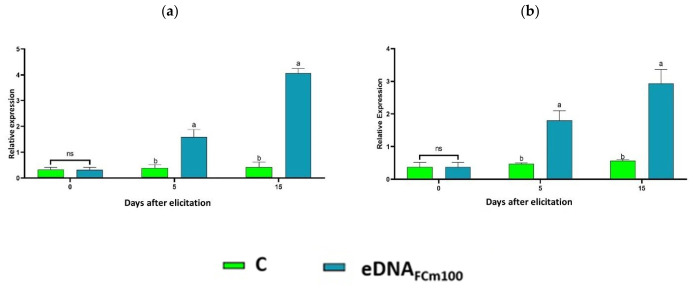
Relative gene expression of *pal3* and *chs* and at 0, 5 and 15 dae in tomato plants treated with eDNA fragments of *Cm*. (**a**) Relative expression of the phenylalanine ammonium lyase (*PAL3*) and (**b**) Relative expression of the chalcone synthase (*CHS*). Average ± standard deviation data of three biological replicates are shown. Equal lower-case letters in each bar for each time indicate a significant statistical difference according to the Tukey test (*p* = 0.05). C, Control plants, eDNA_FCm100_, DNA fragments from *Cm* at 100 µg mL^−1^. ns, not significantly different.

The expression patterns of the *pal3* and *chs* (genes related to the phenylpropanoids biosynthetic pathway and stress response in plants) were analyzed at 0, 5 and 15 dae in the treatments C and eDNA_Fcm100_ (Figure 7). eDNA treatment at 100 µg mL^−1^ was chosen because of its performance in the earlier evaluated variables, being the treatment with the most intense effect on both plant elicitation and biostimulation variables measured previously. The *pr1a* gene expression was also evaluated, but only at 15 days after elicitation with FCm100 treatment (Supplementary Figure S1), showing a higher expression as a result of the treatment. In general, both gene expressions were induced as a result of the application of the treatments with respect to C. This suggests, as expected, that secondary metabolism was induced by this treatment, particularly the synthesis of phenolic compounds, as shown in the previous section. In the case of *pal3* (Figure 7a) and *chs* (Figure 7b), the highest expression levels occurred at 15 dae.

### 2.4. Analysis of Antioxidant Enzyme Activity in Tomato Plants Treated with eDNA Fragments of the Pathogen Cm

**Figure 8:**
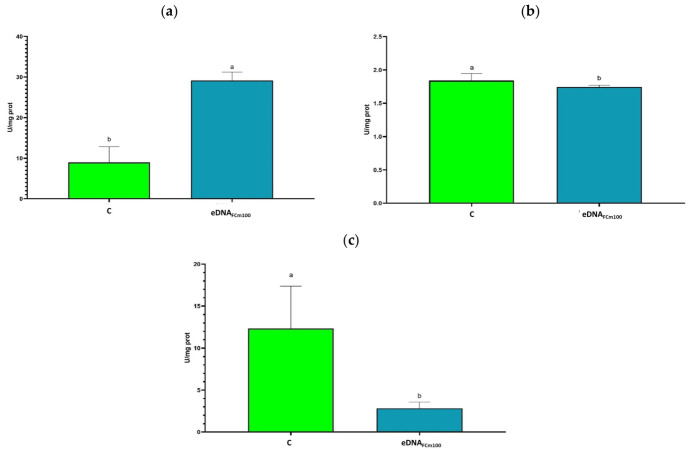
Enzyme activity of PAL, SOD and CAT at 15 dae in tomato plants eDNA fragments of *Cm*. (**a**) Enzyme activity of the phenylalanine ammonium lyase (PAL). (**b**) Enzyme activity of superoxide dismutase (SOD). (**c**) Enzyme activity of the catalase (CAT). Average ± standard deviation data of three biological replicates are shown. Different lower-case letters in each bar for each time indicate a significant statistical difference according to the Tukey test (*p* = 0.05). Symbology: C, Control plants. FCm100, DNA fragments from *Cm* at 100 µg mL^−1^.

The effect of eDNA treatment on the antioxidant enzyme activities related to stress response was evaluated only in the FCcm100 treatment, because, as mentioned above, in the previous variables evaluated in this study, in general, that treatment displayed the best biostimulant responses. As shown in Figure 8a, on the one hand, the treatment with eDNA caused a significant increase in PAL activity at 15 dae. On the other hand, SOD and CAT activities showed a significant decrease compared to the C group (Figure 8b,c).

## 3. Discussion

Biostimulants have recently arisen as common products for agricultural use; their principal goals are to increase productivity to meet the population’s feeding needs and optimize the resource use efficiency while reducing the environmental impact of production systems [13]. Biostimulants are molecules that modulate plants’ metabolism depending on the species. Extracellular DNA has been shown to affect plant metabolism, focusing plant energy on specific biological tasks such as the activation of the plant immune system [19]. This is a positive effect when crops are exposed to pests and pathogens, causing a decrease in the infection symptoms, and resulting in an increment in the final yield. On the other hand, it may be expected that elicitation causes limitations on plant growth or development because of the highly explained growth-defense trade-offs [20].

In this work, extracellular DNA from the pathogen *Clavibacter michiganensis* was applied onto tomato plants, and growth and immune variables were monitored at different times of the experiment. Almost all the morphological, biochemical, and molecular variables evaluated displayed a similar response: an increment with statistical differences from C starting at 5 days after the elicitation for treatment concentrations of 50 and 100 µg mL^−1^ and no differences or a decrease for the concentration of 150 µg mL^−1^. These results recall a common hormetic response in plants treated with stress factors, consisting of a biphasic response to a stimulus depending on its concentration, expecting positive responses (eustress/biostimulation) at low doses but negative responses (excessive toxic elicitation/distress) at higher doses [21]. Thus, in the present work, the higher treatment (eDNA_FCm150_) showed no differences compared to C at morphological variables and a negative effect on root development because of the accumulation of distress in plants.

In a recently published work, plant responses to non-self DNA were characterized from a transcriptomic approach [22]. A high gene expression reprogramming was reported: among others, an upregulation of genes related to salicylic acid (SA) and abscisic acid responses, biotic stress and systemic acquired resistance (SAR), also an enrichment by upregulated genes related to photosynthesis and light harvesting. These results suggest that non-self DNA causes effects on plants through the SA-signaling. The foliar application of SA has been reported to improve photosynthesis rate, carbon dioxide assimilation and stomatal conductance [23], causing an increment in plant growth [24,25,26] and the increment of quality and quantity of secondary metabolites production [27], like the results obtained by the application of *Cm* eDNA in the present work. Additionally, we have reported an upregulation of the *pal3* gene expression that functions as a marker of the phenylpropanoid pathway activation by plant stress responses.

On the other side, SAR has caused a limiting effect on plant growth in specific conditions, for example, Mauch et al. [28] engineered *A. thaliana* plants with SA levels 20-fold above wild type, which showed a strongly dwarfed phenotype, possibly caused by a depletion of the chorismate pools of the chloroplast, as the authors discussed. Similarly, in this work, while the *Cm* pathogen DNA was applied in a higher dose (150 µg mL^−1^), the effect on plant growth is lower or not observed, which suggests that at higher doses it may become harmful for plant growth. As evidence accumulates, SA is identified as an essential player in plant growth regulation, although its action is not fully described. Altered SA levels in plants have resulted in abnormal growth phenotypes, and whilst the level is higher, it induces a stunted stature of plants [29]. The effects vary between stem, fruit and root growth, number of leaves, flowering, leaf area, etc., it seems to be a relation with the regulation of cell division and expansion.

The application of eDNA_FCm100_ and eDNA_FCm50_ showed clear biostimulation effects on plants from day 15 after the elicitation for morphological variables and day 5 after the elicitation for secondary metabolites production. These results contrast with an earlier study where *Capsicum annuum* plants were treated with a mixture of phytopathogen fungi fragmented DNA at 20, 60 and 100 µg mL^−1^, and the plants showed a decrease in height from 14 days after the elicitation and root development at the end of the assay [8]. However, the effect on the synthesis of defense metabolites was presented in this study, showing an increment from the early days after the elicitation. This may highlight the importance of evaluating different concentrations in each plant species, as well as the DNA origin and application form.

Additionally, phenological variables have been monitored in plants, obtaining significant differences between treatments. Specifically, we can observe early flowering events as an effect of eDNA_FCm50_ and eDNA_FCm100_ treatments. It is known that flowering is regulated by several environmental factors and can also be affected by stress; specifically, abiotic stress can induce or accelerate flowering [30]. Abiotic stress has been reported to induce flowering in some plants, but researchers linked the effect to infection of reproductive organs or the overproduction of phytohormones such as auxins by bacteria or other organisms [31]. Here, we observed the effect of the plant sensing danger signals and responding to them by triggering defense responses on the flowering process and the ability of the plant to produce fruits. Flowering and fructification are important agronomic variables that can, to some extent, predict the yield of each plant and treatment, but also the timing of production. Interestingly, eDNA_FCm50_ caused an earlier fruit set, and this may represent input savings for producers.

In another study, the enzymatic activity of PAL, CAT and SOD was determined after non-self DNA application to tomato plants, causing an increment in these immune markers from the first hours and a decrement at 10 days after, as shown here [19]. CAT and SOD represent the earlier responses and efforts of the plant to balance the oxidative burst activated after the detection of danger signals (such as a PAMP). This may explain why a reduction was observed at 15 dae, but the activation effect was maintained for phenols and flavonoids, which represent the late responses of plants to danger. This response was also reported by Agati et al. [32] when *Ligustrum vulgare* plants were exposed to UV light and saline stress, and antioxidant enzymatic activity was activated as an early response, which decreased at 12 days of the treatment, and the concentration of flavonoids increased at the same time as a late response. In this context, the downregulation of SOD and CAT enzymatic activities at 15 dae may suggest an earlier activation and later autoregulation of the enzyme.

The effect of eDNA of different origins (self and non-self) on plants has been evaluated and reported to be highly different, with a phylogenetic-closeness-dependent dependence [19,22,33]. Self DNA effect has been more related to jasmonic acid signaling and has shown different intensity in their responses, which may suggest the result of the development of different immune responses highly specific to the sensed stimulus. But it is still unclear if all non-self DNA produces similar effects or if DNA extracted from pathogenic organisms may activate specific PAMP responses different than ecologically neutral non-self DNA or self-DNA. This is an important matter to assess to clarify the mechanisms of detection and discerning of the DNA origin from the plant.

**Figure 9:**
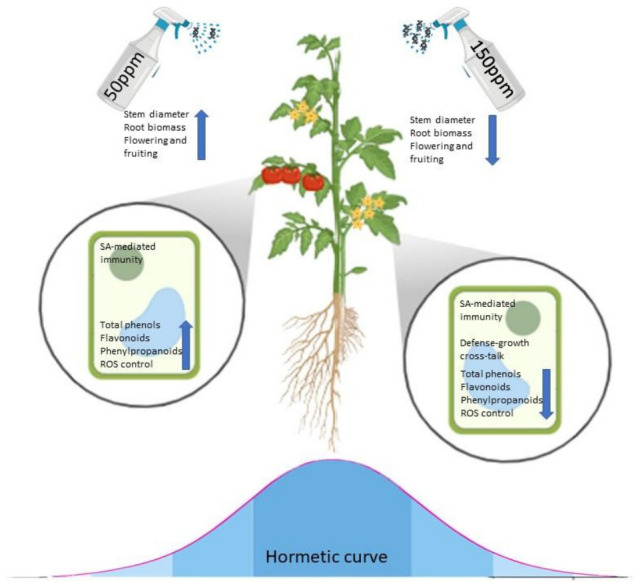
Suggested mode of action and effects of the application of FCm to tomato plants.

In Figure 9, a diagram of the proposed response to fragmented *Cm* DNA of tomato plants is shown. The results reported here suggest a dose-dependent effect of the treatment likely mediated by SA based on the results obtained in oxidative burst and its control, transcription and metabolic reprogramming of genes related to phenylpropanoid pathway and defense, and changes in growth and development of the plants. Notably, the differences between doses suggest a hormetic response of the Cm eDNA treatments evaluated and a defense-growth trade-off for higher doses. This work highlights the importance of doses of *Cm* eDNA to identify effects of elicitation and/or biostimulation when applied to crops.

Finally, *Capsicum* plants’ responses have been reported to increase hydrogen peroxide and antioxidant enzyme activity at 3–5 days after elicitation with salicylic acid, hydrogen peroxide and chitosan [34], all of which are involved in the PAMPs signaling pathway. This characterization of the response that a specific plant species has to a specific stimulus may help researchers to develop stress management strategies for the agricultural industry in a way that biostimulation and elicitation maintain only their positive effects, and plant stress never reaches the distress zone. Hormetic studies using eDNA as plant biostimulants are necessary to define more accurate applications of these molecules in sustainable crop production. Future studies should consider evaluating the intensity of photosynthesis caused by the eDNA treatments, changes in phytohormones related to plant growth (auxins, gibberellins, citokinins, etc), as well as the activities of other antioxidant enzymes, such as several peroxidases, and even the changes in hydrogen peroxide content, to unravel in more detail the eDNA effects when studying biostimulation in plants. In addition, the gene expression associated with plant defense, i.e., PR genes, transcription factors like WRKY, among others, should also be studied to get a better understanding of this phenomenon when using eDNA in plant biostimulation. It would also be interesting to evaluate the stability of eDNA in soil, taking into account the results of previous work in which the eDNA of *Fusarium oxysporum* was stable at least during seven days in soil [9]; however, this aspect remains to be explored. Moreover, profitable strategies for the scale-up of eDNA production will also be necessary to develop.

## 4. Materials and Methods

### 4.1. Biological Material

Tomato (*Solanum lycopersicum* L.) seedlings, Guanacaste variety (*Seminis* seed company, St. Louis, MO, USA), with 10–12 true leaves, were used. The 10-day acclimatization process was carried out, placing them in a greenhouse bag with sterile Sunshine 3M substrate under greenhouse conditions (average temperatures of 15–19 °C at night and 26–35 °C during the day and average humidity of 70–85%). An automated drip irrigation system was established, which watered the plants daily according to the general Steiner solution [35].

The pathogen used in this study was isolated from Guanajuato state in Central México, affecting tomato plants with bacterial canker disease. This pathogen was identified as *Clavibacter michiganensis* (*Cm*) using a specific PCR strategy reported by Sousa Santos et al. [36]. The strain is stored at the laboratory of molecular phytopathology in the Universidad Politécnica de Guanajuato, labeled as strain Cmm12C3.

### 4.2. Extraction and Fragmentation of Genomic DNA from Clavibacter michiganensis

To obtain the DNA fragments, *Cm* was first grown in ATCC Medium 763: NBY medium (Lifeasible, Shirley, MA, USA) incubated at 28 °C for 24 h, then centrifuged at 8800× *g* for 7 min to recover the cell sediment, the supernatant was discarded, and DNA extraction was performed based on the CTAB protocol [37] with some modifications: biological tissue were finely ground using mortar and pestle and liquid nitrogen, 500 mg of the frozen powder was mixed with 1 mL of extraction buffer (100 mM tris (IBI Scientific, Dubuque, IA, USA), 1.4M NaCl (J. T. Baker, Phillipsburg, PA, USA), 20 mM EDTA (J. T. Baker, Phillipsburg, PA, USA) pH 8.0, 2% Cetyltrimethylammonium bromide (CTAB, Sigma-Aldrich, St. Louis, MO, USA) and 0.3% β-mercaptoethanol (Gibco, Walthman, MA, USA) at 60 °C and vortexed during 1 min. The samples were incubated at 60 °C for 15 min, allowed to cool, and then 500 µL of chloroform: isoamyl alcohol (24:1) (HiMedia Laboratories, Mumbai, India) was added. The mix was vortexed again and centrifuged at 17,500× *g* for 15 min. at 4 °C. The upper aqueous phase was collected in a new tube, and the nucleic acids were precipitated with ½ volume of 5M NaCl (J. T. Baker, Phillipsburg, PA, USA) and 2 volumes of cold isopropanol (IBI Scientific, Dubuque, IA, USA). The tubes were left overnight at −20 °C and then centrifuged at 17,500× *g* for 15 min at 4 °C. The supernatant was discarded, and the obtained pellet was washed with 75% ethanol (Hycel, Guadalajara, Mexico) and dried at room temperature, then resuspended with TE buffer and stored at −40 °C until used. Once the DNA from *Cm* was obtained, concentration and purity were evaluated by spectrophotometric measure of absorbance at 260, 230 and 280 nm in a NanoDrop 2000/2000 c from Thermo Scientific (Waltham, MA, USA). Fragmentation was performed using a Vibra-Cell Ultrasonic processor VCX130 (Sonics & Materials, Inc., Newtown, CT, USA) at a frequency of 20 KHz, 10 W, and an amplitude of 50% per second for 20 min. The size of the fragments was then determined by electrophoresis in 1.2% agarose gel, 1 Kb plus weight marker, 1X gel intercalator, at 80 V for 30 min. Once fragments between 100 and 1500 bp were verified, DNA concentrations were adjusted to 50, 100, and 150 µg mL^−1^ for storage at −40 °C until use.

### 4.3. Application of eDNA Fragments from the Pathogen Cm to Tomato Plants (Plant Elicitation)

After 10 days of acclimatization, the plants were sprayed with eDNA fragments from the pathogen *Cm* (eDNA_FCm_) at concentrations of 50, 100, and 150 µg mL^−1^, respectively, for each treatment. Spraying was carried out at the point of dripping according to Mejía-Teniente et al. [34]. This means that the volume of sprayed solution depended on the tissue area of each plant, the application was uniformly applied at all leaves, and the applied volume increased with the growth of plants. C plants were sprayed with water in the same way as the treated plants. Twenty-four plants were used per treatment, with three replicates. For the determination of phenols, flavonoids, gene expression and enzymatic activity of two leaves from the middle canopy position of four plants per repetition per treatment were sampled. Each repetition was collected on the described days and stored at −80 °C until further processing. These plants were excluded from the other variables to avoid bias in measures.

### 4.4. Measurement of Morphological and Phenological Variables of Plants

The height and diameter of the plant stems were measured in centimeters at 0, 5, 15, and 30 days after application of eDNA fragments of the pathogen *Cm* (days after elicitation, dae). In the case of height, a tape measure was used, measuring from the base to the apex of the plant, while a vernier caliper was used to measure stem diameter.

The length, width, and weight of the root biomass of the plants were determined in six plants per each of the three repetitions of each treatment at 30 dae using a flexometer and a vernier caliper. The dry weight of the root of the different treatments was determined, drying the tissue of the plant using an oven at 60 °C for a time of 48 h until it reached a constant weight, as checked with a weighing scale. The remaining plants were kept in culture conditions until 69 dae, when the assay was finished. Flowering and fructification were monitored on days 21 (only flowering), 28, 42, 58 and 69 dae and reported as flowers and set fruits per plant.

### 4.5. Determination of Phenols and Total Flavonoids Content

The determination of phenolic compounds for all the evaluated treatments was carried out at 0, 1, 5, 15, and 30 dae, according to Mejía-Teniente et al. [34]. The tissue was ground with liquid nitrogen, mortar, and pestle until obtaining a fine powder, and 50 mg of the powder was homogenized in 2.5 mL of absolute methanol, protected from light, with constant stirring at 150 rpm, 20 °C for 24 h. After this time, samples were centrifuged for 10 min at 9000× *g*, and the supernatant was recovered and stored at −20 °C in the dark. After extraction, the total phenol content was determined by the Folin–Ciocalteu method, adapted for use in microplates. The reaction mixture consisted of 20 μL of the extract, 230 μL of distilled water, and 125 μL of Folin–Ciocalteu reagent; the sample was homogenized and left to stand for 5 min, and 625 μL of 20% NaCO_3_ was added. The mixture was homogenized and left to stand for 2 h in the dark. After the resting time, 250 μL were taken to place on the microplate, and then the absorbance at 760 nm was determined on a Thermo Scientific Multiskan GO spectrophotometer. The number of total phenols was expressed in micrograms equivalents of gallic acid per gram of fresh weight. For the determination of total flavonoids, it was performed according to Iqbal et al. [38]. For this method, 125 μL of the extract was mixed with 25 μL of 10% (*w*/*v*) aluminum chloride, 25 μL of 1M potassium acetate, 375 μL of 80% methanol, and 700 μL of distilled water. This mixture was homogenized and allowed to stand for 30 min at room temperature. Subsequently, the absorbance at 415 nm was determined on a Thermo Scientific Multiskan GO spectrophotometer. Total flavonoids were expressed in micrograms of quercetin equivalents per gram of fresh weight. Three biological replicates were carried out in the study for these measurements.

### 4.6. Analysis of Gene Expression Associated with Stress Response in Plants

For the extraction of total RNA, plant tissue from the collected leaves was ground in liquid nitrogen, and the TRIzol^R^ Reagent method (Ambion (Kaufungen, Germany), Life Technologies (Waltham, MA, USA)) was used, following the manufacturer’s methodology. The integrity of the extracted RNA was checked on agarose gel electrophoresis, and its purity and concentration were assessed using a NanoDrop. Complementary DNA (cDNA) synthesis was performed using the Clontech PCR-Select cDNA subtraction package (BD Biosciences, San Jose, CA, USA), which uses oligo (dT) as primers. The cDNA of all samples was diluted to 400 ng uL^−1^ and stored at −20 °C for qPCR until further analysis.

Gene expression analysis was performed using RT-qPCR of two genes reported as indicators of immunity induction in tomato plants: phenylalanine ammonium lyase (*PAL3*, GenBank accession number X78269) and chalcone synthase (*CHS*, GenBank accession number FJ705842.1). As a housekeeping gene control, actin (*ACTIN*, Gene Bank accession number AB199316.1) was used.

For the amplification of these genes in the treated tomato plants, primers used were previously reported by Rodríguez-Calzada et al. [39]; 200 ng of cDNA was used to perform qPCR, using Maxima SYBR Green qPCRMaster Mix Thermo Scientific, in the C100 Touch Thermal Cycler device, BIORAD CFX96 Real Time System brand. Amplification conditions for both measured genes were 5 min at 94 °C, 40 cycles of 94 °C for 1 min, 55 °C for 1 min, and 65 °C for 5 s; Amplification conditions for *ACT* were 5 s at 94 °C and 40 cycles of 5 s at 94 °C and 30 s at 57 °C. The primers used for each amplification were: forward: 5′-CAAAAGATAATGCTAAAACTGTA-3′ and reverse: 5′-CCCATTTTCCAGCGAGAT-3′ for *PAL3*, forward: 5′- CCAAGGACTTGGCTGAGAAC-3′ and reverse: 5′-TATCGGGGACAAGAGTTTGG-3′ for *CHS* and forward: 5′-AGTTGCCCCAGAAGAACACC-3′ and reverse: 5′-CCACCACCTTGATC TTCATG-3′ for *ACTIN*.

This analysis was performed on treated tomato plants at 0, 5 and 15 dae and only in plants treated with eDNAFcm100 and C. Three biological replicates were analyzed in this study for all treatments. Relative gene expression was determined using the ΔΔ^Ct^ method [39].

### 4.7. Determination of Enzymatic Activity

For enzymatic activity determination, an enzymatic extract was obtained from C and from the group with a higher global response to the application: eDNAF_cmm100_ at 15 dae. The procedure consisted of using 300 mg of frozen leaves powder, directly homogenized with 2 mL of extraction buffer (0.05 mM Potassium phosphate, pH 7.8) for superoxide dismutase and 1 mL of extraction buffer for phenylalanine ammonium-lyase and catalase, then vortexed for two minutes. Subsequently, the extract was centrifuged at 9000× *g* for 15 min, and the supernatant was collected to obtain the enzymatic extracts.

#### 4.7.1. Phenylalanine Ammonium-Lyase (PAL)

PAL activity was estimated following the protocol of [40] with slight modifications. 20 μL of the enzymatic extract was mixed with 230 μL of reaction buffer (0.1 M Borate, 10 mM phenylalanine; pH 8.8). The mixture was incubated at 40 °C for 1 h and stopped with 50 μL of 5 N HCl. The absorbance was measured at a wavelength of 290 nm. The activity was quantified as mmol of cinnamic acid formed per gram of protein and expressed as units per milligram of protein (U mg^−1^). Each treatment was repeated three times.

#### 4.7.2. Catalase (CAT)

CAT activity was quantified following the protocol of Afiyanti and Chen [41]. Then, 0.1 mL of the enzymatic extract was mixed with 2 mL of reaction buffer (Potassium phosphate 50 mM, pH 8), and 0.2 mL of 100 mM H_2_O_2_ was added to a quartz cell to immediately measure the absorbance at 240 nm every minute for 6 min to calculate the rate of H_2_O_2_ decreased by the spectrophotometer. One unit of CAT activity is equal to 1 μmol/min equivalent of H_2_O_2_ consumed per milliliter per minute. Each treatment was repeated three times.

#### 4.7.3. Superoxide Dismutase (SOD)

One unit of SOD is referred to as the amount of enzyme needed to exhibit 50% dismutation of the superoxide radical. SOD activity was calculated by the 50% inhibition of nitro blue tetrazolium [42]. The enzymatic extract (0.05 mL) was mixed with 1.5 mL of the reaction buffer, 0.3 mL of 0.1 mM EDTA-NA_2_, 0.3 mL of 0.13 M methionine, 0.3 mL of 0.75 mM nitroblue tetrazolium, 0.3 mL of 0.02 mM riboflavin, and 0.25 mL of distilled water. The mix was exposed to UV light, controlling the same amount of light for each tube for 15 min., and absorbance was measured at 560 nm.

#### 4.7.4. Protein Determination

Finally, determination of protein concentration was performed for all samples to express enzymatic activities as units per milligram of protein (U mg^−1^). The Bradford protocol was used [43]. Briefly, 0.02 mL of enzymatic extract was mixed with 0.23 mL of Bradford solution in a 96-well plate and allowed to rest in the dark for 20 min. Then the absorbance was measured at 595 nm, and the concentration of protein was determined using serial dilutions of a bovine serum albumin solution as a protein standard curve.

### 4.8. Statistical Analysis

The biostimulation effect of eDNA fragments from the pathogen *Cmm* on *Solanum lycopersicum* tomato plants was evaluated using a completely randomized block design. To determine statistical differences in all variables, two-way analysis of variance (ANOVA) and Tukey’s test, *p* < 0.05, were performed on GraphPad Prism 10.2.0 software.

## 5. Conclusions

Fragmented eDNA from *Cm* has been shown to cause biostimulation effects in tomato plants when applied at 50 and 100 µg mL^−1^. The results showed an increment in plant production of biomass and activation of antioxidant enzyme activity and second metabolite biosynthesis, which have been recognized as plant immune markers. This strategy may represent an effective application in the field with low environmental and health risks to crop producers and final consumers that might contribute to the development of more sustainable agricultural systems.

## Data Availability

The original contributions presented in this study are included in the article/Supplementary Material. Further inquiries can be directed to the corresponding authors.

## Supplementary Materials

The following supporting information can be downloaded at: https://www.mdpi.com/article/10.3390/plants15111599/s1. Figure S1: Gene expression of pr1a at 15 days after elicitation using Cm eDNA at 100 µg mL^−1^.

## Abbreviations

The following abbreviations are used in this manuscript:

PAMP	Pathogen-associated molecular pattern
PTI	PAMP-triggered immunity
MAPK	Mitogen-activated protein kinase
ROS	Reactive oxygen Species
ETI	Effector-triggered immunity
DAMP	Damage-associated molecular pattern
eDNA	Extracellular DNA
RLCKs	Receptor-like cytoplasmic kinases
EF-Tu	Elongation factor Tu
PR	Pathogenesis-related proteins
Cm	*Clavibacter michiganensis*
dae	Days after elicitation
FCm	DNA fragments from the pathogen *Clavibacter michiganensis*
PAL	Phenylalanine ammonia-lyase
Chs	Chalcone synthase
CAT	Catalase
SOD	Superoxide dismutase
SA	Salicylic acid
SAR	Systemic Acquired Resistance
