# Supplementary figures and images for: Biostimulation of Tomato Plants (Solanum lycopersicum L.) Using Fragmented Extracellular DNA from Clavibacter michiganensis
Source: Plants (Basel). 2026 May 22;15(11):1599. doi: 10.3390/plants15111599 (PMC13259155; doi:10.3390/plants15111599)

Relative Expression

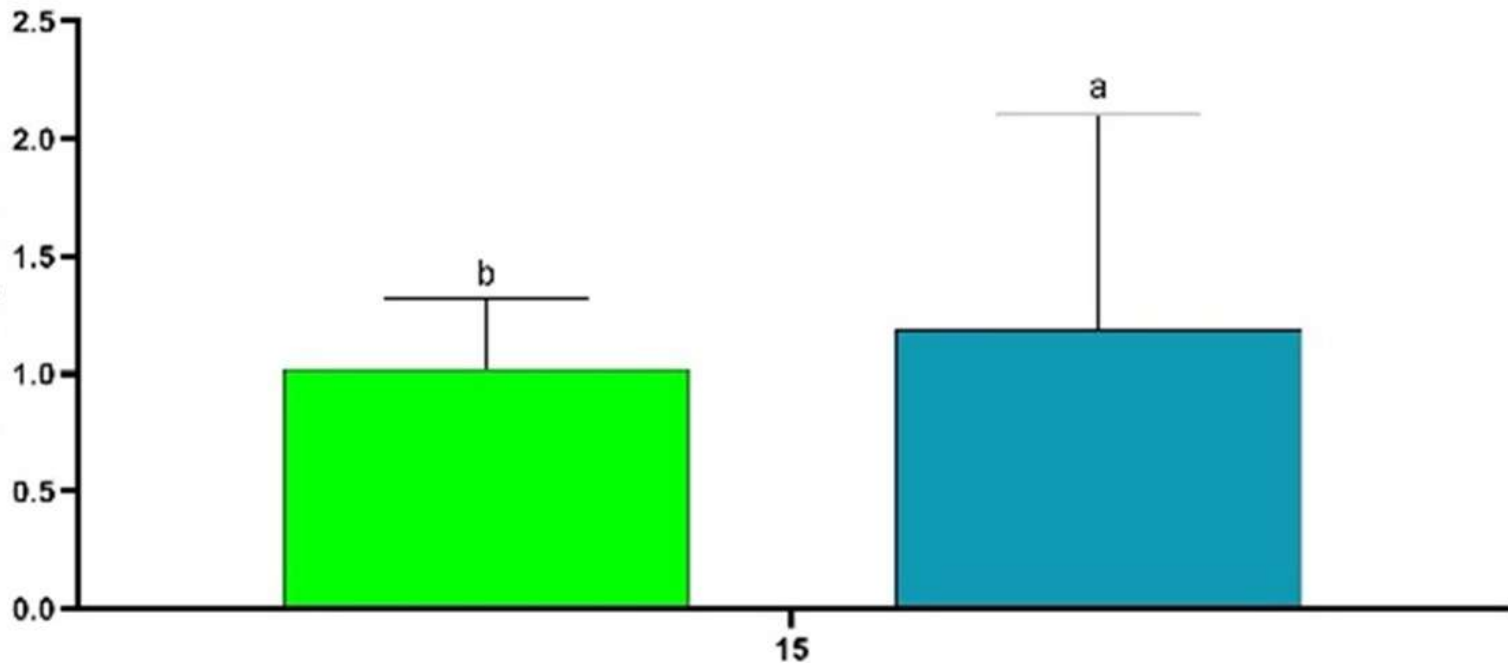

Days after elicitation

C

eDNA<sub>FCM100</sub>

Supplement: Supplementary file 1 [file plants-15-01599-s001.zip › plants-4309006-supplementary.pdf]
